# Effects of remifentanil and propofol on distant organ lung injury in an ischemia–reperfusion model

**DOI:** 10.1515/med-2021-0381

**Published:** 2021-11-08

**Authors:** Orhan Kanbak, Burcu Aydoğan, Tülin Gümüş

**Affiliations:** Anesthesiology and Reanimation Department, Ankara City Hospital, Mutlukent mh. 2023 sok. No: 13 Çankaya, Ankara 06800, Turkey; Anesthesiology and Reanimation Department, İstanbul Metin Sabancı Baltalimanı Bone Diseases Education and Research Hospital, İstanbul, Turkey; Anesthesiology and Reanimation Department, Ankara City Hospital, Ankara 06800, Turkey

**Keywords:** oxidative stress, ischemia–reperfusion injury, anesthetics, intravenous

## Abstract

Our aim was to evaluate lung injury due to oxidative stress and antioxidant activity levels in an infrarenal ischemia–reperfusion model and to compare prevention effects of single and combined use of propofol and remifentanil. In this study, a total of 40 adult Wistar Albino rats were randomly divided into five groups of eight rats as SHAM, physiological saline, intraperitoneal propofol, remifentanil, and propofol and remifentanil groups. Blood and tissue samples were obtained after 80 min of reperfusion. The malondialdehyde (MDA) level, a measure of lipid peroxidation, was measured in lung tissue samples and red blood cells; additionally, total oxidant status and total antioxidant capacity of lung tissues were measured and histopathological examination was performed. Distant organ (lung) injury developed due to lower extremity ischemia–reperfusion was created by infrarenal aortic clamping. The lipid peroxidation product MDA and total oxidant levels were increased, but there was insufficient antioxidant protection both in the lung tissues and red blood cells. While propofol prevented this injury consistent with its proposed antioxidant properties; no protective effect of remifentanil was observed. On the contrary, it showed oxidative stress increasing effect. This study concluded that the antioxidant effect of propofol was suppressed by remifentanil in the case of combined use.

## 1 Introduction

It has been shown that ischemia–reperfusion (I/R) injury is not only limited to the tissue exposed to ischemia but the other distant organs are also affected by some mediators and toxic products like oxygen radicals, cytokines, complement proteins, prostaglandins and thromboxane released to the systemic circulation [1,2,3]. One of the distant organs mostly affected by I/R injury is the lungs. Lung injury that occurs following I/R leads to significant postoperative mortality and morbidity. In the case of lung injury caused by I/R, the findings are hypoxemia, pulmonary hypertension, reduced lung compliance and nonhydrostatic pulmonary edema and the severity of the disease may vary from a temporary subclinical condition to a serious condition, resulting in acute respiratory distress syndrome (ARDS) [4,5,6,7].

Various studies have shown the protective effects of antioxidant compounds against distant organ injury developing after acute I/R injury. Although there have been a large number of drugs or substances that have been studied for their antioxidant effects and are being used for that purpose, anesthetic substances have a different and important place among them [8,9,10,11,12,13,14,15]. One of these anesthetic substances that have been greatly emphasized is propofol. Due to its structural characteristics, it has free radical scavenging properties. As propofol is highly lipid-soluble, it particularly accumulates on lipophilic membranes, which are very sensitive to oxidative injury, and may increase or prevent the decrease in antioxidant capacity [16,17,18,19].

Remifentanil is the only opioid metabolized to its inactive metabolites by nonspecific plasma and tissue esterases. It is widely distributed in plasma, red blood cells and interstitial structures. Myocardial protective effects of remifentanil against I/R injury have been reported in several studies [20,21,22].

Our aim was to evaluate distal organ lung injury in terms of oxidative stress and antioxidant activity levels in an infrarenal ischemia–reperfusion (I/R) model and to compare the antioxidant and lung injury prevention effects of single and combined use of propofol and remifentanil as preconditioning.

## 2 Materials and methods

The study was performed in Erciyes University Experimental Research and Application Center in accordance with the “Guiding Principles for Research Involving Animals and Human Beings,” by the approval of the Institutional Animal Care and Use Committee of Erciyes University (Approval number: 11/62). In this study, 40 male Wistar albino rats, 5 months old, weighing 200 ± 40 g were used. During the experiments, all rats were kept in a 10 h light/14 h dark cycle, at a room temperature of 24 ± 2°C, and were fed with standard pellet and tap water.

Rats were divided randomly into five experiment groups, each containing 8 rats. Group 1 (SHAM) rats underwent laparotomy, closed, and after 100 min their tissue and blood samples were obtained; they were not treated with any substances during the experiment. Following laparotomy, Group 2 (PS) rats were infused with physiological saline, in the same amount of drug volume that the other groups received. While Group 3 (PRO) and Group 4 (REM) rats received 50 mg kg^−1^ h^−1^ propofol and 20 μg kg^−1^ min^−1^ remifentanil, respectively; Group 5 (PRO + REM) rats received 20 μg kg^−1^ min^−1^ remifentanil and 50 mg kg^−1^ h^−1^ propofol infusion starting 10 min before ischemia and continued for 30 min during ischemia. After 60 min of reperfusion, their tissue and blood samples were obtained.

### 2.1 Ischemia–reperfusion model

On the day of the study, the rats were weighed and anesthetized using intraperitoneal 1.6 mg kg^−1^ urethane. Following anesthesia, the abdomen of rats was dissected and the infrarenal abdominal aorta was explored; by placing an atraumatic vascular clamp, ischemia was achieved. In order to prevent fluid and heat loss, the abdominal region of the rat was closed using gauze soaked with warm physiological saline under a heating lamp and the body temperature of the rats was kept at 37 ± 1°C. The drugs were given intraperitoneally using an infusion pump; the infusion was started 10 min before ischemia and continued during ischemia. After 30 min of ischemia, the clamp was removed; restoring blood flow and perfusion were re-established at the distal tissues. After 60 min of reperfusion, intracardiac blood samples and lung tissue samples were obtained for histopathological and biochemical evaluations.

The lung tissue samples were washed with physiological saline and were divided into two parts: the part separated for histological examination was put in 10% formaldehyde, and the other part, the lung tissue sample, was stored at −70°C for biochemical measurements. In blood samples, hemorheological parameters were tested within 2 h, the remaining blood was centrifuged at 2,500 rpm for 10 min to separate plasma and red blood cells (RBC). RBC were washed three times with physiological saline and both RBC and plasma were stored at −20°C for biochemical measurements.

### 2.2 Biochemical measurements

The measurement of malondialdehyde (MDA), a product of lipid peroxidation, is based on the spectrophotometric reaction of MDA with thiobarbituric acid (TBA), forming a pink colored complex showing maximum absorbance at 532 nm wavelength [23].

The method developed by Ohkawa et al. was used in the assessment of MDA levels in lung tissue samples [24]. The supernatant obtained by centrifugation of tissue homogenates (1/10 w/v) was used: 0.1 mL of supernatants was put into a glass tube with a stopper, and 0.1 mL of sodium dodecyl sulfate (SDS), 0.75 mL of acetic acid, 0.75 mL of TBA and 0.3 mL of distilled water were added and the mixture was stirred. The tightly closed tubes were brewed in a boiling water bath for 60 min. Then, 0.5 mL of distilled water and 2.5 mL of *n*-butanol/pyridine mixture were added to the tubes, and the tubes were cooled with tap water and mixed with a vortex. Following extraction, the absorbance of the pink-colored organic phase that was formed on the upper part of the tubes, which were centrifuged at 4°C at 4,000 rpm for 15 min, was measured with a spectrophotometer at 532 nm (Unicam Helios Beta), against blind control that was formed with distilled water in the same way. Quantitation was done using a standard curve. Tissue MDA levels were calculated (nmol/ml) per milligram protein (nmol MDA/mg protein).

In lung tissue homogenates, total oxidative stress (TOS), total antioxidant capacity (TAC) and oxidative stress index (OSI) were measured using commercial kits (Rel Assay Kit Diagnostics® Gaziantep, Turkey) by Erel’s specific fully automated measurement method [25,26]. The total oxidative stress measurement method is based on the oxidation of bivalent ferrous iron to trivalent ferric iron by the oxidants present in the sample. In an acidic medium, ferric iron ions form a colored complex with xylenol and are read spectrophotometrically at a wavelength of 660 nm.

The experiment is calibrated by H_2_O_2_, and the values are expressed as μmol Trolox equivalent/mg protein. The total antioxidant capacity method is based on the measurement of the characteristic color formed by total antioxidants of the sample with more stable 2,2′-azino-bis (3-ethylbenz-thiazoline-6-sulfonic acid) (ABTS) radicals at 530 nm and is expressed as mmol Trolox equivalent/mg protein. The oxidative stress index was calculated by the following formula: OSI = [(TOS, μmol Trolox equivalent units/mg protein)/(TAC, mmol Trolox equivalent units/mg protein)].

### 2.3 Histological methods

The collected tissue samples from the right inferior lobe were fixed in 10% neutral formalin solution for 72 h at room temperature. The fixed tissues were examined under a light microscope, and paraffin blocks were formed. The prepared blocks were cut using a microtome (Leica SM 2000, Germany), and 4–5 μm thick sections were mounted on polylysine-coated slides. The sections were stained using hematoxylin–eosin to assess the structural changes, examined under light microscopy, and assessed in terms of histopathological parameters.

### 2.4 Statistical analysis

Shapiro–Wilk test was used to test normality, and one-way ANOVA test was performed for inter-group comparisons. If there is a significant difference, *post-hoc* Scheffe test was used to reveal the group that made the difference. Data are presented as mean ± SD.

All analysis was done using SPSS Statistics 20.0 (IBM-SPSS Inc., Chicago, IL) software program. A *p* value <0.05 was accepted as the significance level.

## 3 Results

### 3.1 Erythrocyte and lung tissue MDA values

The levels of MDA, an oxidative stress indicator and lipid peroxidation product, in lung tissue samples and RBC are presented in Table 1.

**Table 1:** MDA levels in lung tissues and RBC

Groups	Erythrocyte MDA (nmol/g Hb)	Lung MDA (nmol/mg protein)
SHAM (*n* = 8)	78.09 ± 2.50^†^	2.66 ± 0.29^†^
PS (*n* = 8)	225.69 ± 5.67*	6.62 ± 0.28*
PROP (*n* = 8)	190.33 ± 4.21*^††^	2.88 ± 0.13^†^**
REM (*n* = 8)	371.44 ± 35.06*^†^	7.89 ± 0.38*^†^
PROP + REM (*n* = 8)	257.44 ± 10.91*	4.39 ± 0.27*^†^

Values are presented as mean ± standard deviation.

*p < 0.0001 in comparison to the SHAM group, **p < 0.005 in comparison to the SHAM group.

^†^ *p* < 0.0001 in comparison to the PS group, ^††^ *p* < 0.005 in comparison to the PS group.

The lung tissue and erythrocyte MDA levels of all I/R-treated groups were significantly higher than that of the SHAM group, while the greatest increase was seen in the remifentanil group (*p* < 0.0001). Erythrocyte and lung MDA levels were significantly lower in the propofol group compared to the saline group, but both values were observed significantly higher in the REM group (*p* < 0.0001). On the other hand, while the lung MDA level was lower in the PROP + REM group than in the PS group, there was no significant difference between the erythrocyte MDA levels.

### 3.2 Total oxidant (TOS) and antioxidant capacity (TAC) levels in lung tissues

Total oxidative stress and total antioxidant capacity of the lung tissues are presented in Table 2.

**Table 2:** Total oxidative stress (TOS) and total antioxidant capacity (TAC) of lung tissues

Groups	Lung tissue TOS (µmol Trolox equivalent U/g protein)	Lung tissue TAC (mmol Trolox equivalent U/g protein)	OSI (AU)
SHAM (*n* = 8)	6.46 ± 0.28^†^	2.63 ± 0.23^†^	2.46 ± 0.15 ^†^
PS (*n* = 8)	10.53 ± 0.24*	1.57 ± 0.16*	6.75 ± 0.74*
PRO (*n* = 8)	7.48 ± 0.15**^†^	4.15 ± 0.27*^†^	1.80 ± 0.10^†^
REM (*n* = 8)	10.63 ± 0.93*	2.53 ± 0.22^†^	4.24 ± 0.63*
PRO + REM (*n* = 8)	8.91 ± 0.29*^†^	3.01 ± 0.10*^†^	2.95 ± 0.09^†^

Values are presented as mean **±** standard deviation.

OSI: oxidative stress index; AU: arbitrary unit, *p < 0.0001 in comparison to the SHAM group, **p < 0.004 in comparison to the SHAM group, ^†^ *p* < 0.0001 in comparison to the PS group.

Total oxidative stress (TOS) and total antioxidant capacity (TAC) of lung tissue samples are expressed in mmol per gram protein. Total oxidative stress and total antioxidant capacity measured in lung tissue samples showed parallelism with the MDA results. While the TOS levels were significantly higher in all I/R-treated groups compared to the SHAM group, the TAC level was lower in the PS group, higher in the PRO and PRO + REM groups (*p* < 0.0001), and no significant difference was observed in the REM group. On the other hand, while TOS levels did not differ in the REM group compared to the PS group, they were significantly lower in the other two groups (*p* < 0.0001).

TAC levels were found to be significantly higher in all three drug-administered I/R groups than in the PS group (*p* < 0.0001).

### 3.3 Oxidative stress index (OSI) values in lung tissue samples

The ratio of total oxidants to antioxidants in lung tissue samples (TOS/TAC) was expressed as the oxidative stress index. When OSI was compared between the groups, the highest OSI value was in the PS group. According to the Scheffe test, all inter-group comparisons were found to be statistically significant (*F* = 150.82, *p* < 0.0001), except SHAM and PRO (*p* = 0.09) and SHAM and PRO + REM (*p* = 0.33) groups.

### 3.4 Histopathological findings

**Figure 1:**
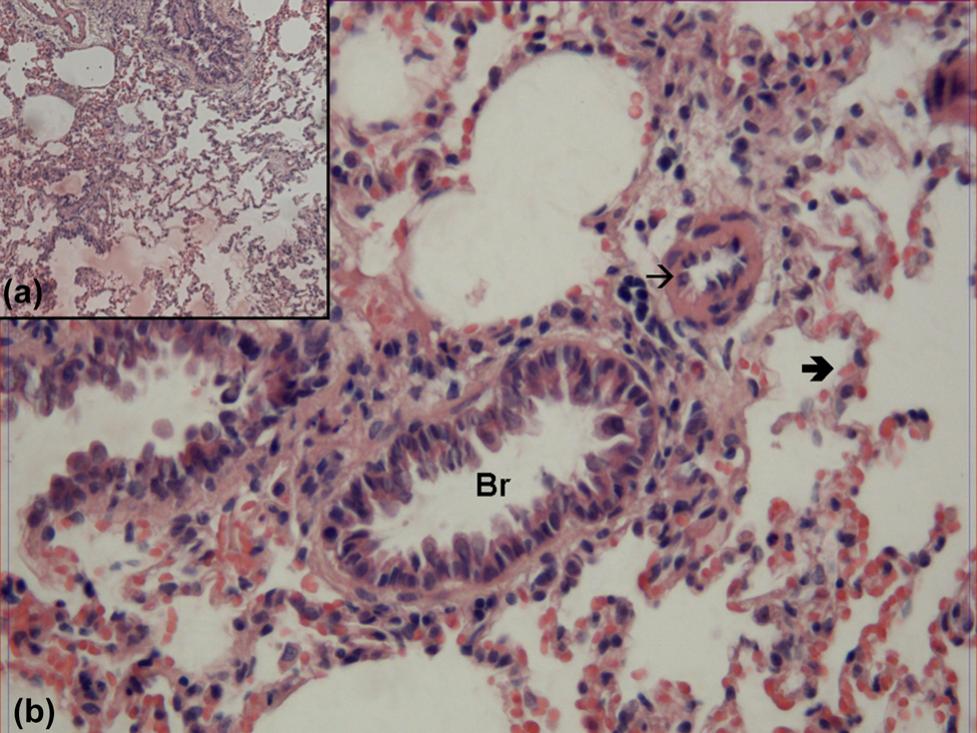
Hematoxylin–eosin-stained lung samples obtained from the SHAM group. Br: bronchioles, 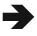: alveoli, →: vessel (hematoxylin–eosin, A: ×100, B: ×400).

In the evaluation of hematoxylin–eosin-stained lung samples obtained from the control group, the bronchi, bronchioles, alveoli and interalveolar septa showed normal structure both by ×100 and ×400 magnification (Figure 1).

**Figure 2:**
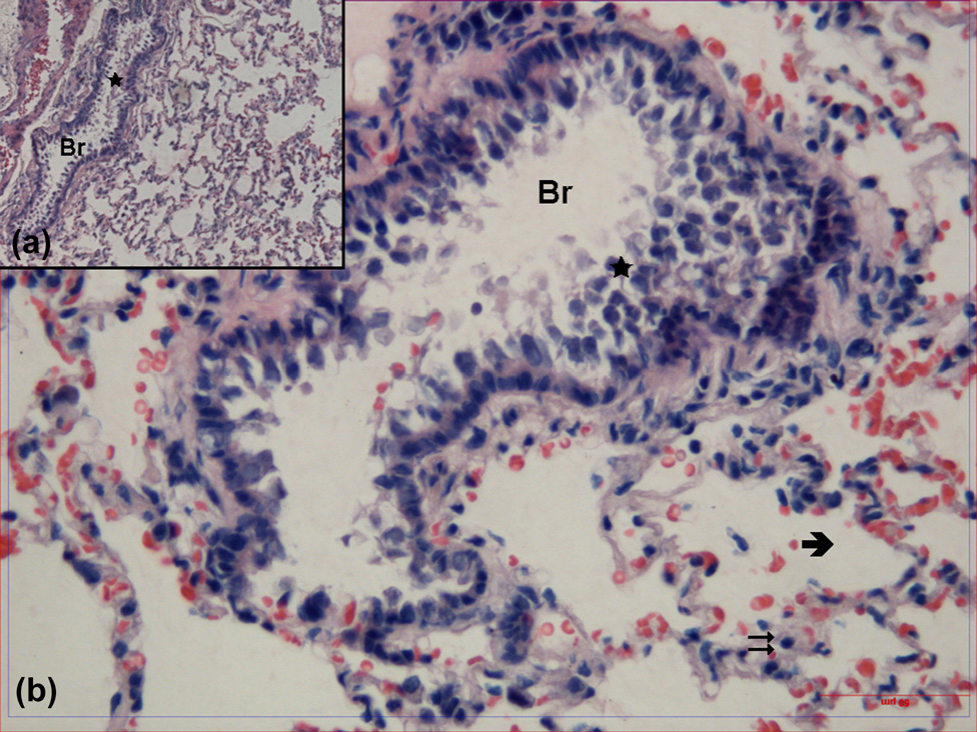
Hematoxylin–eosin-stained lung tissue samples obtained from the group treated with physiological saline. Br: bronchioles, 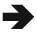: alveoli, 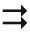: interalveolar septa, 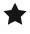: cell debris (hematoxylin–eosin, A: ×100, B: ×400).

The cell debris composed of epithelial cells was observed in the bronchiole lumen of tissue samples obtained from the PS group. In high magnification imaging, it was seen that while the structure was protected in some areas, the epithelial layer was disrupted in some areas and some epithelial cells with pyknotic nuclei fall into the lumen. Alveoli and interalveoler septa were also thickened (Figure 2).

**Figure 3:**
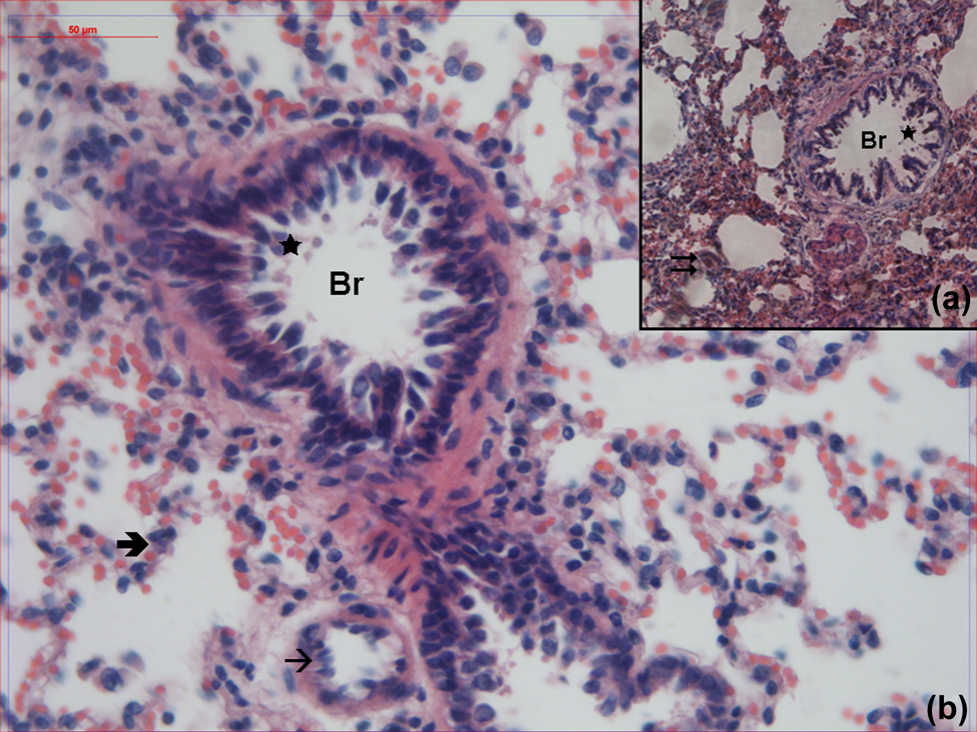
Hematoxylin–eosin-stained lung samples obtained from the propofol-treated group. Br: bronchioles, 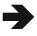: alveoli, 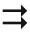: interalveolar septa, 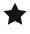: cell debris, →: vessel (hematoxylin–eosin, A: ×100, B: ×400).

In the samples obtained from the propofol-treated group, epithelization was quite normal in the bronchioles. It was observed that epithelial debris in bronchiole lumens was less significant compared to the PS group by high magnification. The thickness of the interalveoler septum was normal and the wall structure was similar to that of the SHAM group (Figure 3).

**Figure 4:**
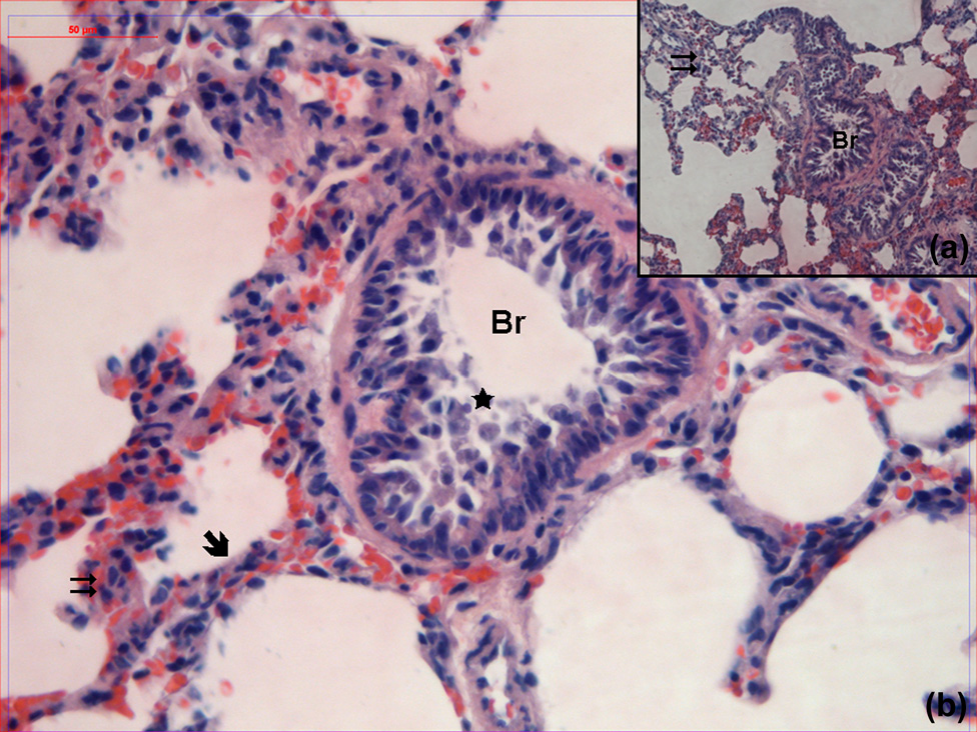
Hematoxylin–eosin-stained lung samples obtained from the group treated with remifentanil. Br: bronchioles, 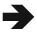: alveoli, 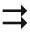: interalveolar septa, 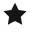: cell debris (hematoxylin–eosin, A: ×100, B: ×400).

When the lung samples obtained from the REM group were evaluated, it was observed that the epithelial cell debris in the lumen of the bronchioles was more significant and the interalveoler septum was thickened (Figure 4).

**Figure 5:**
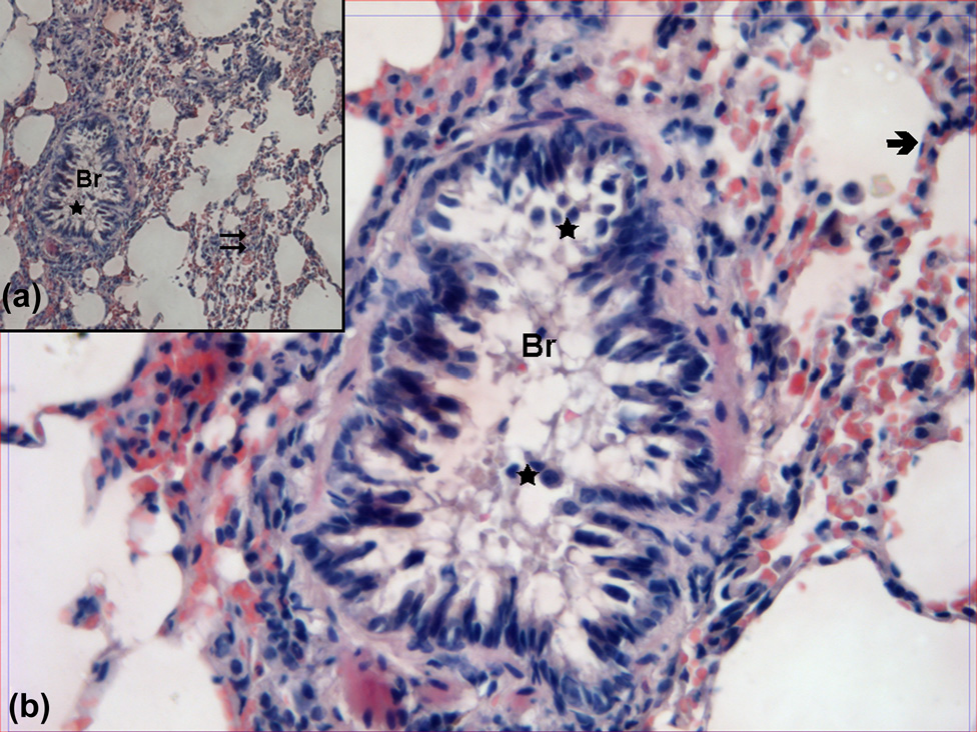
Hematoxylin–eosin-stained lung samples obtained from the group treated with remifentanil plus propofol. Br: bronchioles, 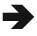: alveoli, 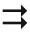: interalveolar septa, 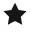: cell debris (hematoxylin–eosin, A: ×100, B: ×400).

In the examination of the tissue samples obtained from the PRO + REM group, it was observed that the cell debris in the bronchiole lumens was extremely significant. Although alveoli showed normal structure in some areas, interalveolar septa were partially thickened (Figure 5).

## 4 Discussion

In our study, experimental ischemia–reperfusion was created with infrarenal abdominal aortic occlusion in rats, and the lungs that were most affected by distant organ damage due to their rich microcapillary system were selected to be examined; MDA, TOS and TAS levels were measured as indicators of oxidative damage. Increased erythrocyte and lung MDA levels in all groups underwent I/R compared to the SHAM group, indicating that pulmonary oxidative injury develops as a result of infrarenal aortic ischemia–reperfusion.

Histological findings also showed a parallel trend to MDA results. While the lung samples obtained from the SHAM group showed a normal structure, the partial destruction of epithelization, cell debris, thickening of alveoli and interalveoler septa were observed in the samples obtained from the PS group are the results of lung tissue injury after ischemia–reperfusion.

Intraperitoneal infusions of propofol, remifentanil and propofol plus remifentanil were started 10 min before ischemia and were continued during ischemia. The application of drugs in this way may also be considered as preconditioning. It has been proposed that antioxidant substances show their effects by preventing the increase in pulmonary microvascular permeability, neutrophil adhesion and accumulation or by binding to free oxygen radicals, thus showing their protective effects against distant organ injury that develop after I/R [10,11,27,28,29] In our study, positive results could only be achieved by propofol, and remifentanil showed no preventive effects on the injury.

It was demonstrated in our study that the lung tissue MDA levels increased in all I/R-treated groups compared to the SHAM group, and ischemia–reperfusion injury was significantly prevented in the propofol-treated group, while combined use of propofol and remifentanil partially prevented I/R injury. The highest level of MDA was observed in the remifentanil group. Remifentanil infusion failed to prevent lungs against lower extremity ischemia–reperfusion injury; on the contrary, the level of oxidative injury was further increased in that group.

Therefore, our study shows that propofol is also effective in preventing distant organ lung injury in rats with infrarenal aortic ischemia. Propofol infusion was started before ischemia at a subanesthetic dose and continued during ischemia in our study. In this regard, it differs from the other ischemia–reperfusion injury prevention models. In a study emphasizing this issue, it was reported that propofol administration as induction was not very effective in terms of protection, but continuous infusion during the operation could reduce the damage [30]. This finding confirms our results.

As well known, due to the weak analgesic effect of propofol, it is used together with tranquilizers, sedatives or various analgesics. One of these combinations is the use of propofol as a hypnotic and remifentanil as an analgesic due to their short duration of action. Therefore, we compared whether remifentanil alone or in combination with propofol infusion is effective in preventing ischemia–reperfusion injury with the effect of propofol alone. Remifentanil is a short-acting opioid that metabolizes to its inactive metabolites by nonspecific plasma and tissue esterases. It is widely distributed in plasma, RBCs and interstitial structures. It has been proposed that remifentanil can suppress the cellular immune response in sepsis and may be protective for lungs, which are one of the earliest affected organs [31]. Some researchers found that opioid analgesics may significantly reduce cytokine response, and propofol and remifentanil suppress TNF-α and IL-6 induction, thus affecting neutrophil migration and adhesion molecule expression [19,32] Recently, studies on the protective effects of remifentanil against intestinal, hepatic and myocardial I/R injury have been reported [33,34]. These studies show that remifentanil prevents I/R damage in the local target organ.

In our study, while propofol alone had a very high antioxidant effect against the lung tissue I/R damage, remifentanil had no protective effect. We observed that remifentanil did not prevent lung injury as a distant organ in infrarenal I/R. In our previous study, Erkılıç et al. [35] compared the protective effects of remifentanil and dexmedetomidine (DEX) on renal I/R injury. We have demonstrated that neutrophil gelatinase-associated lipocalin (NGAL) levels and histopathological findings reflected protection by DEX against renal I/R injury while remifentanil was not as effective as DEX.

In our study, it was observed that the antioxidant effect of propofol was suppressed by remifentanil in the PROP + REM group animals. Observations obtained as a result of the examination of histological sections also support our biochemical findings. Indeed, the cell debris consisting of epithelial cells in the bronchiolitis lumen was prominent and the interalveolar septum thickness increased in the remifentanil group.

As a result of ischemia–reperfusion, along with the injuries in both local and distant organs, RBC in the blood circulation are also affected. As RBC are cells carrying oxygen to the tissues, they are among the main structures exposed to oxidative stress, hence, free oxygen radicals. These oxygen radicals may lead to lipid peroxidation, and changes in enzymes, other proteins, and, especially, the hemoglobin molecule [36]. All these changes negatively affect the oxygen transport functions of RBC. This situation would certainly affect the other tissue and organs, not in the ischemic region.

Therefore, in our study, along with oxidative injury in the lungs, RBC MDA levels that could contribute to the injury were measured. The RBC MDA levels were expressed as nmol per gram hemoglobin and show parallelism to values measured in lung tissues. The erythrocyte MDA levels were higher in I/R-treated groups in comparison to that in the SHAM group. The highest MDA levels were again found in the remifentanil and propofol plus remifentanil groups. The lowest MDA levels among the three groups that were treated with anesthetic substance infusion were observed in propofol-treated rats. Another *in vitro* study proposed that propofol shows protective effects against oxidative injury in RBC, partially protects their mechanic or rheological properties, and therefore, may be beneficial in decreasing surgical procedure injury [37].

Oxidative stress-related rheological changes in RBC negatively affect their functions, and thus, the blood flow and oxygenation of various tissues and organs. The reason for the single or combined use of different anesthetics is their different effect mechanisms and increased protective effect against oxidative injury; in this way, alternative protocols can be developed in the selection of anesthetic substances in surgical procedures. However, as our study demonstrated, combined use of some anesthetics may not be an appropriate choice. In our study, besides showing no protective effects against oxidative injury, remifentanil seems to increase oxidative injury in the red blood cells. This might both be due to its vulnerability to being metabolized by circulation enzymes because of its ester bond and its binding to proteins [38]. To our knowledge, there are no studies on whether remifentanil is bound by RBC or affected by them or not.

## 5 Conclusion

In conclusion, distant organ lung injury is developed by lower extremity ischemia–reperfusion by infrarenal aortic clamping. The lipid peroxidation product MDA and total oxidant levels increase both in lung tissues and red blood cells, and antioxidant protection remains insufficient. While propofol infusion at subanesthetic doses prevents this injury by showing an antioxidant effect, remifentanil shows no protective effect; on the contrary, it shows oxidative stress increasing effect. It was also observed in the histological examination that propofol decreased negative effects of I/R on tissues, while remifentanil was not effective as propofol. In the group that was treated with both agents, remifentanil maintained its effects and perhaps suppressed the positive effects of propofol. Although these two anesthetic substances with different anesthetic structures and properties have been preferred to be used together, it was concluded that in the case of combined use of both agents the antioxidant effects of propofol are suppressed. Therefore, in oxidative stress-associated cases such as abdominal or lower extremity surgeries, it should be kept in mind that I/R may cause distant organ lung injury, and there is a need for new studies to investigate the effect of remifentanil on I/R damage on distant organs, especially on lung injury.
